# The Association of Depression, Anxiety, and Quality of Life with Sexual Function in Young-to-Middle-Aged Stroke Patients: A Case-Control Study

**DOI:** 10.1177/10538135261468010

**Published:** 2026-07-24

**Authors:** A Ozakgul, Z Tulek, K Kılıçaslan, S Erden Melikoğlu, H Kaya

**Affiliations:** 1 Department of Fundamentals of Nursing, Florence Nightingale Faculty of Nursing, Istanbul University-Cerrahpasa, İstanbul Üniversitesi-Cerrahpaşa Florence Nightingale Hemşirelik Fakültesi Avcilar Yerleşkesi, Üniversite Mahallesi, Üniversite Caddesi, Avcilar, İstanbul, Türkiye; 2 Department of Medical Nursing, Florence Nightingale Faculty of Nursing, Istanbul University-Cerrahpasa, Istanbul, Türkiye

**Keywords:** Stroke, sexual dysfunction, ASEX, quality of life, rehabilitation

## Abstract

**Background:**

Despite its impact, sexual health remains a neglected component of rehabilitation for younger stroke patients.

**Aim:**

To evaluate sexual dysfunction prevelance in stroke patients and examine its association with health-related quality of life and psychological distress.

**Methods:**

This case-control study included 30 stroke patients and 30 matched controls. Data were collected using the Arizona Sexual Experience Scale (ASEX), Stroke-Specific Quality of Life Scale (SSQOL), and Hospital Anxiety and Depression Scale (HADS).

**Results:**

Patients had significantly higher total ASEX scores (19.3 ± 6.6 vs. 13.0 ± 3.1, *p* < 0.001), and a higher prevalence of dysfunction than controls (*p* = 0.004), reporting a significant decline in sexual satisfaction (7.5 to 4.1, *p* < 0.001). Sexual function was significantly associated with the SSQOL Energy, Thinking, and Language domains (*p* < 0.05), and HADS-Depression (*r* = 0.450, *p* = 0.013), but not with functional state.

**Strengths and Limitations:**

Strengths include a matched control group. Limitations include a small sample and cross-sectional design, potential selection bias from a high refusal rate, and restricted psychological variance due to clinical exclusions.

**Conclusion:**

Sexual dysfunction represents a multifaceted challenge for young-to-middle-aged stroke patients, appearing more closely linked to perceived quality of life domains (cognitive, energy, emotional well-being) than to motor deficits. Sexual health may be affected even in patients with low physical disability.

**Clinical Implications:**

Rehabilitation should address holistic needs. Healthcare professionals could benefit from incorporating sexual health screenings into routine care, considering the management of fatigue and depressive symptoms to support long-term well-being.

## Introduction

Stroke is a major health condition that profoundly impacts an individual's bio-physiological, psychological, and sociocultural dimensions. Post-stroke sequelae frequently extend into intimate life, resulting in reduced sexual function and satisfaction (Dusenbury et al., 2017). As sexuality is a fundamental component of human experience and a basic need, these difficulties can adversely influence multiple life domains, thereby diminishing overall quality of life (QoL) (Park et al., 2015; Latella et al., 2024). Despite its significance, sexuality remains a sensitive and often unaddressed topic in clinical care. Recent research emphasizes the necessity of systematic sexual health evaluations, noting that tailored support for patients and their partners significantly improves recovery outcomes (Zhang et al., 2025).

Global and national data underscore the severity of the stroke-related complications. Stroke is the second leading cause of death worldwide and in Türkiye, ranking as a primary cause of long-term disability (Arsava, 2017; Feigin et al., 2020). The resulting impairments vary depending on the neuroanatomical site of the lesion. For instance, lesions in the medial frontal cortex are often associated with hyposexuality, while temporal lobe or bilateral amygdala damage may lead to hypersexuality (Geller & Wong, 2025). Furthermore, left-sided lesions are frequently linked to post-stroke depression, which can manifest as severe apathy, social isolation, and a subsequent decline in both motor and sexual activity (Hess et al., 2017).

Literature indicates that the prevalence of sexual dysfunction varies widely, typically occurring in approximately 35% to 70% of stroke patients, and may persist for years (Dusenbury et al., 2017; Stein et al., 2013; Geller & Wong, 2025; Latella et al., 2024). Common manifestations include decreased libido and frequency of intercourse for both genders, reduced lubrication and orgasmic dysfunction in women, and erectile or ejaculatory failure in men (Steiner & IsHak, 2017). These problems arise from a complex interplay of organic factors—such as sensory-perceptual deficits that reduce pleasure—and psychosocial barriers, including fear of recurrence, reduced self-esteem, and altered body image. Beyond these overt impairments, the “invisible” burden of stroke—specifically neuropsychiatric symptoms like depression and anxiety—plays a critical role in inhibiting sexual desire and satisfaction (Greenberg et al., 2017; Steiner & IsHak, 2017). Furthermore, emerging evidence suggests that sleep disturbances, which rank among the most common non-motor outcomes of stroke, can further disrupt the physiological and hormonal balance necessary for healthy sexual function (Ozkan et al., 2025). Qualitative evidence suggests women often perceive stroke-related physical changes as a direct threat to their femininity (Lever & Pryor, 2017). Cognitive and communication difficulties further compound these issues. While motor recovery is often the primary focus of rehabilitation, research indicates that sexual function can remain significantly impaired even in patients with mild or no physical disability (Tamam et al., 2008). This suggests a complex relationship between functional capacity, invisible symptoms, and sexual health that requires deeper investigation (Greenberg et al., 2017).

Sociocultural factors are also among the barriers that prevent patients from expressing their sexual concerns. In traditional societies such as Türkiye and other Eastern cultures (Dusenbury et al., 2025), sexual health is generally considered a taboo topic (Çengelci Özekes et al., 2022), particularly in the context of serious illness (Er & Yildirim, 2019). This cultural context, together with healthcare professionals’ prioritization of mobility-related issues over sexuality and time constraints, often hinders comprehensive assessment and counseling for stroke patients (Richards et al., 2016).

Nurses, as primary members of the rehabilitation team, are uniquely positioned to identify these frequently overshadowed challenges. Evidence suggests that individualized interventions targeting sexual health can yield meaningful benefits for patients (Brandão et al., 2025). However, descriptive data on post-stroke sexuality in our population remains limited. Therefore, utilizing a comparative baseline approach, the present study was designed to evaluate the prevalence of sexual dysfunction following stroke and to examine its specific associations with psychological distress and quality of life domains to advocate for more holistic neurorehabilitation practices.

## Methods

### Aim of the Study

This study utilized a cross-sectional, descriptive case–control design to evaluate the extent of sexual dysfunction and its relationship with disability, psychological distress, and quality of life following stroke. This methodology was selected to establish a comparative baseline against a healthy cohort, isolating the specific impact of the stroke event from general age-related factors. By examining a comprehensive range of clinical and psychological variables, this design provides a robust evidence base for integrating sexual health into traditional mobility-focused neurorehabilitation practice.

### Sample and Setting

The sample consisted of stroke patients who presented to the outpatient clinic of the neurology department of a university hospital between October 2020–April 2023 met the inclusion criteria, and volunteered to participate. The control group was composed of adults who presented to the general neurology outpatient clinic of the same hospital for non-serious complaints (e.g., headache, dizziness), had no known neurological or systemic diseases, and who were matched to the stroke group by age and sex. Inclusion criteria for all participants (patients and controls) were being an adult aged 18–65 years, being married or having a regular sexual partner, and having no difficulty understanding or answering the questions. To minimize the confounding effects of primary psychiatric disorders on sexual function, strict exclusion criteria were applied to both groups. Participants were excluded if they had a known history of depression or were currently using antidepressant medications. Additionally, the Hospital Anxiety and Depression Scale (HADS) was utilized as a screening instrument during sample selection; individuals scoring above the clinical cut-off points were excluded to ensure the study population did not present with significant clinical anxiety or depression. Patients were included if they had a confirmed stroke diagnosis for at least six months and no history of recurrent stroke, and excluded if they had severe functional impairment, defined as a Modified Rankin Scale score above 4. This case-control study included 30 stroke patients and 30 matched controls.

### Data Collection

Individuals who met the study criteria were informed about the aim of the research, and written informed consent was obtained. Data were collected through face-to-face interviews in a private outpatient room without the presence of companions. The following tools were used:

#### Patient Information Form

Developed by the researchers, this form assessed sociodemographic and clinical characteristics. A 0–10 visual analog scale was used to assess satisfaction with sexuality before and after stroke.

#### Arizona Sexual Experience Scale (ASEX)

The ASEX (male and female versions) is a six-point Likert-type scale with five items assessing sex drive, arousal, vaginal lubrication/penile erection, ability to reach orgasm, and satisfaction from orgasm. Total scores range from 5 to 30, with higher scores indicating worse sexual function (McGahuey et al., 2000; Soykan, 2004). The Turkish validity and reliability study reported a Cronbach's alpha of 0.88 (Soykan, 2004). In the present study, the Cronbach's alpha coefficient was 0.911. Sexual dysfunction was identified using the original diagnostic criteria of the scale. While some literature in Türkiye suggests a lower threshold (≥11) for broad screening purposes to maximize sensitivity, we opted for the standard three-fold diagnostic criteria to ensure higher clinical specificity. A participant was categorized as having sexual dysfunction if they met at least one of the following criteria: (1) a total score of ≥19, (2) a score of ≥5 on any individual item, or (3) a score of ≥4 on any three items. This robust approach allows for the detection of both global sexual impairment and severe domain-specific dysfunction, providing a more precise comparison between the stroke and control groups.

#### Stroke Spesific Quality of Life Scale (SSQOL)

Developed by Williams et al. (1999), the SSQOL assesses quality of life after stroke. The Turkish validity and reliability study reported a Cronbach's alpha of 0.97 (Hakverdioğlu Yönt & Khorshid, 2012). In the current study, Cronbach's alpha was found to be 0.952. The scale contains 49 items across 12 subscales, scored on a five-point Likert format. Higher scores indicate better quality of life.

#### Hospital Anxiety and Depression Scale (HADS)

Developed to screen for symptoms of anxiety and depression in individuals with physical illnesses (Zigmond & Snaith, 1983), the scale consists of 14 items: 7 for anxiety (HADS-A) and 7 for depression (HADS-D). HADS was used to examine how sub-clinical levels of anxiety and depression correlate with sexual dysfunction and health-related quality of life. In the Turkish validation study, the cut-off scores were reported as 7 for depression and 10 for anxiety (Aydemir et al., 1997). Cronbach's alpha values were 0.829 for anxiety and 0.792 for depression in our sample.

#### Modified Rankin Scale (mRS)

The mRS is widely used to assess functional status after stroke, scoring disability from 0 to 6. Scores ≤2 indicate good functional status, whereas scores >4 indicate severe impairment.

### Statistical Analysis

Statistical analyses were performed using IBM SPSS Statistics (v.31.0). Descriptive statistics, including means, standard deviations, medians and frequencies, were used to present the data. As the data were not normally distributed, non-parametric tests were employed. The Mann-Whitney U test and Kruskal-Wallis test were used for comparisons between two and three independent groups, respectively. For within-group comparisons of pre-test and post-test scores, the Wilcoxon Signed-Rank test was applied. Categorical variables were analyzed using the Chi-Square test or Fisher's Exact test, where appropriate. Correlations between continuous variables were evaluated using Spearman's rank correlation coefficient. For all analyses, statistical significance was considered as *p* < 0.05.

### Ethical Considerations

Ethical approval was obtained from the ethics committee of the hospital (date:8 Sept 2020, no:59491012-604.01.02-A-39). Written informed consent was obtained from all participants prior to data collection.

## Results

### Sociodemographic and Clinical Characteristics

During the recruitment process, 20 individuals who otherwise met the inclusion criteria (the majority of whom were female) declined to participate. The primary reason provided was discomfort regarding the sensitivity of the topic, resulting in a final sample of 30 patients. The sociodemographic and clinical characteristics of the participants are presented in Table 1. Patients were slightly older than the controls (48.9 ± 9.8 vs. 44.6 ± 9.9 years, *p* = 0.057). The mean age of the patient group was 48.9 years, representing a relatively young stroke population within the active adult age range. Compared to the control group, patients had significantly lower education level (*p* = 0.007) and employment rates (*p* = 0.003). The groups were comparable regarding gender, marital status, and income levels. In terms of clinical profiles, the majority of patients (90%) had experienced an ischemic stroke, with 86.7% having a good functional state (mRs < 3). As expected, patients demonstrated significantly higher rates of comorbidities (80.0% vs. 10.0%, *p* < 0.001) and medication use (80.0% vs. 6.7%, *p* < 0.001) compared to controls. Psychological assessment showed significantly higher scores for both anxiety (*p* = 0.015) and depression (*p* = 0.014) in the patient group (Table 1).

**Table 1.** Sociodemographic and Clinical Characteristics of the Participants.

	Pt with Stroke	Control		
	*n* (%)	*n* (%)	*Z*/*χ*^2^	*p*
Sex				
Female	15 (50)	15 (50.0)	0.000	1.000
Male	15 (50)	15 (50.0)		
Education				
<High school	18 (60.0)	6 (20.0)	10.050	**0**.**007**
High school	6 (20.0)	11 (36.7)		
University	6 (20.0)	13 (43.3)		
Marital status				
Married	28 (93.3)	26 (86.7)	**—**	0.671
Having a partner	2 (6.7)	4 (13.3)		
Working status				
Working	13 (43.3)	24 (80.0)	8.531	**0**.**003**
Not working*	17 (56.7)	6 (20.0)		
Income				
More than or equal to expenses	21 (70.0)	25 (83.3)	1.491	0.222
Less than expenses	9 (30.0)	5 (16.7)		
Comorbidity				
Yes	24 (80.0)	3 (10.0)	29.697	**<0**.**001**
No	6 (20.0)	27 (90.0)		
Multicomorbidity				
Yes	15 (50)	1 (3.3)	16.705	**<0**.**001**
No	15 (50)	29 (95.7)		
Continuous use of medication				
Yes	24 (80.0)	2 (6.7)	32.851	**<0**.**001**
No	6 (20.0)	28 (93.3)		
Smoking				
Yes	16 (53.3)	12 (40.0)	1.071	0.301
No	14 (46.7)	18 (60.0)		
Alcohol				
Yes	4 (13.3)	6 (20.0)	0.480	0.488
No	26 (86.7)	24 (80.0)		
Type of stroke				
Ischemic	27 (90.0)	—		
Hemorrhagic	3 (10.0)	—		
Functional state (*X* ± SD)(range) (M)	1.4 ± 0.8 (0–3) (1.0)	—		
Good/moderate (mRs < 3)	26 (86.7)	—		
Poor (mRs ≥ 3)	4 (13.3)	—		
Post-stroke deficit	15 (50.0)	—		
Post-stroke aphasia	7 (23.3)	—		
Urinary incontinence	1 (3.3)	—		
Fecal incontinence	0 (0.0)	—		
	X ± SD (range) (M)	X ± SD (range) (M)	*Z*	*p*
Age	48.9 ± 9.8 (25–64) (49.5)	44.6 ± 9.9 (26–64) (42.5)	−1.902	0.057
Body mass index (BMI)	28.4 ± 4.8 (20.5–40.2) (27.9)	24.2 ± 2.6 (19.4–31.3) (27.2)	−3.674	**<0**.**001**
HADS-Anxiety	6.5 ± 4.1 (0–16) (7.0)	4.1 ± 4.3 (0–18) (4.0)	−2.435	**0**.**015**
HADS-Depression	5.6 ± 3.5 (0–11) (7.0)	3.4 ± 3.2 (0–10) (3.0)	−2.464	**0**.**014**

Note: Mann-Whitney U, chi-square and Fisher's chi-square tests were used. mRs: Modified Rankin Scale, HADS: Hospital Anxiety and Depression Scale. * Not working included housewives (*n* = 10), retired (*n* = 3), unemployed participants or patients who could not return work after stroke (*n* = 12).

### Stroke-Specific Quality of Life (SSQOL) Outcomes

The profile of health-related quality of life among stroke survivors is summarized in Table 2. The mean total SSQOL score for the patient group was 3.6 ± 0.8. Analysis of individual domains revealed that patients experienced varying levels of problems across the 12 dimensions of the scale. The most profound problems—represented by the lowest mean scores—were observed in the Energy, Thinking, Mood, and Personality domains. Conversely, the domains of Vision, Self-care, and Language remained relatively preserved within this cohort (Table 2).

**Table 2.** Mean Subscale Scores of the Stroke-Specific Quality of Life (SSQOL) Scale in Stroke Patients.

SSQOL Subscales	X ± SD	Range	Median
Energy	2.5 ± 1.2	1.0–5.0	2.3
Family roles	3.4 ± 1.3	1.0–5.0	3.0
Language	4.3 ± 1.1	1.0–5.0	5.0
Mobility	3.8 ± 1.0	1.8–5.0	3.9
Mood	3.3 ± 1.4	1.0–5.0	3.1
Personality	3.2 ± 1.3	1.0–5.0	3.0
Self care	4.4 ± 1.1	1.0–5.0	5.0
Social roles	3.5 ± 0.9	1.6–5.0	3.3
Thinking	3.1 ± 1.2	1.0–5.0	3.0
Upper extremity functions	4.1 ± 1.3	1.0–5.0	5.0
Vision	4.3 ± 1.1	1.0–5.0	5.0
Work/productivity	3.8 ± 1.2	1.0–5.0	4.0
Total	3.6 ± 0.8	2.1–4.9	3.6

Note. SSQOL: Stroke-Specific Quality of Life.

### Prevalence of Sexual Dysfunction

**Figure 1.**
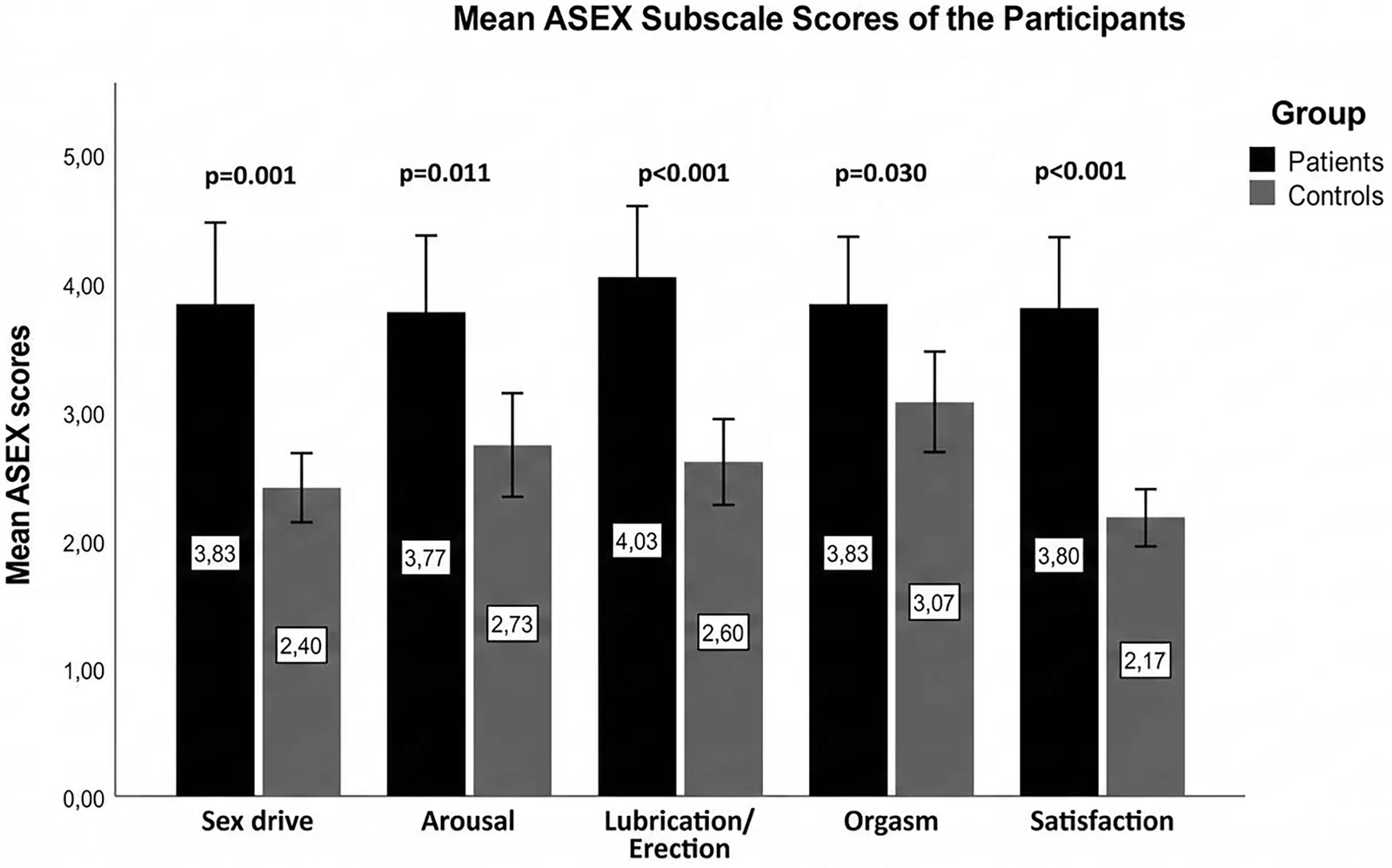
Comparison of mean ASEX subscale scores between patients and controls.

Sexual function evaluation via ASEX revealed significantly higher total scores in the patient group (19.3 ± 6.6 (median 19.0) vs. 13.0 ± 3.1 (median 13.0), *Z* = −3.871, *p* < 0.001), indicating greater dysfunction. Sub-dimension analysis showed that patients scored significantly higher in all domains (Figure 1). The prevalence of sexual dysfunction (based on the cut-off value of ASEX) was markedly higher in patients than in controls (72.0% vs. 28.0%, *χ*^2^ = 8.297, *p* = 0.004) (Data not shown in Tables).

**Figure 2.**
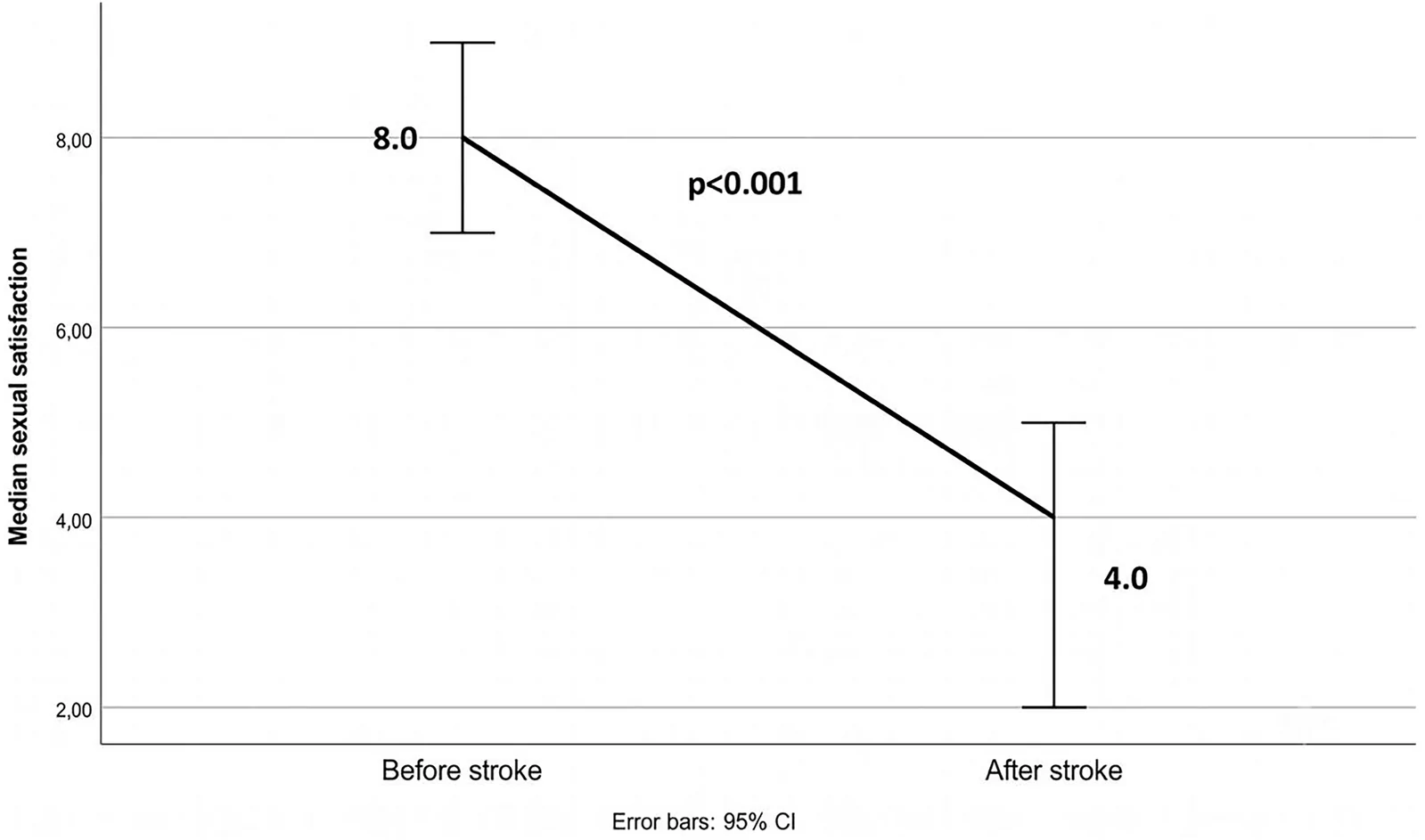
Sexual satisfaction before and after stroke.

In addition to ASEX, patients’ self-reported sexual satisfaction was assessed using a Visual Analog Scale (VAS) ranging from 0 to 10. A significant decline in subjective satisfaction was observed post-stroke, dropping from a pre-stroke mean of 7.5 ± 2.6 to 4.1 ± 2.9 (*Z* = −4.118, *p* < 0.001) (Figure 2).

### Factors Associated with Sexual Dysfunction

Sub-group analyses were conducted to identify factors associated with sexual dysfunction within the patient cohort. Higher ASEX scores—indicating greater dysfunction—were observed in patients with lower education level compared to those with university-level education (20.9 ± 6.9 vs. 14.2 ± 2.9, *χ*^2^ = −5.518, *p* = 0.063), and in the lower-income group compared to those with higher income (22.6 ± 8.3 vs. 17.9 ± 5.4, *Z* = −1.747, *p* = 0.081), though these trends did not reach statistical significance. Clinical factors also played a significant role; the presence of depressive symptoms was associated with significantly higher ASEX scores (22.2 ± 7.1 vs. 15.5 ± 3.6, *Z* = −2.790, *p* = 0.005) (Table 3).

**Table 3:** Mean ASEX Scores According to Patients’ Sociodemographic and Clinical Characteristics.

	X ± SD (median)	*Z*/*χ*^2^	*p*
Sex			
Female	21.1 ± 6.3 (20.0)	−1.518	0.129
Male	17.4 ± 6.6 (15.0)		
Education			
<High school	20.9 ± 6.9 (21.5)	5.518	0.063
High school	19.3 ± 6.5 (17.0)		
University	14.2 ± 2.9 (13.0)		
Working status			
Working	17.5 ± 5.4 (15.0)	−1.343	0.179
Not working	20.6 ± 7.3 (20.0)		
Income			
More than or equal to expenses	17.9 ± 5.4 (16.0)	−1.747	0.081
Less than expenses	22.6 ± 8.3 (24.0)		
Functional state (mRs)			
Good/moderate (mRs < 3)	19.6 ± 6.1 (19.0)	−0.734	0.463
Poor (mRs ≥ 3)	17.0 ± 10.3 (16.0)		
Post-stroke deficit			
Yes	19.3 ± 7.7 (19.0)	−0.104	0.917
No	19.3 ± 5.6 (19.0)		
Post-stroke aphasia			
Yes	19.4 ± 8.5 (14.0)	−0.123	0.902
No	19.2 ± 6.2 (19.0)		
Comorbidity			
Yes	19.9 ± 6.4 (20.0)	−1.273	0.203
No	16.5 ± 7.3 (14.5)		
Smoking			
Yes	17.1 ± 6.9 (14.5)	−1.938	0.053
No	21.7 ± 5.5 (21.0)		
Anxiety			
Anxious (HADS-A ≥ 10)	20.4 ± 7.6 (20.0)	−0.344	0.731
Non-anxious (HADS-A < 10)	18.9 ± 6.4 (19.0)		
Depression			
Depressive (HADS-D ≥ 7)	22.2 ± 7.1 (24.0)	−2.790	**0**.**005**
Non-depressive (HADS-D < 7)	15.5 ± 3.6 (14.0)		
		*r*	*p*
Age		0.228	0.226
Pre-stroke sexual satisfaction	—	0.031	0.869
Body mass index	—	0.050	0.793

Note: Mann-Whitney U, Kruskal-Wallis, and Spearman correlation tests were used.

Correlation analysis revealed distinct relationships between psychological state, quality of life, and sexual function. Depression scores were positively correlated with total ASEX scores (*r* = 0.450, *p* = 0.013) and sub-dimensions of arousal, orgasm, and satisfaction (*p* < 0.05). Notably, sexual function was not correlated with anxiety or functional state (Table 4).

**Table 4.** Correlations of ASEX Scores with Functional State, Anxiety, and Depression in Stroke Patients.

	mRs		HADS-A		HADS-D	
ASEX	*r*	*p*	*r*	*p*	*r*	*p*
Sex drive	0.091	0.633	−0.044	0.818	0.337	0.069
Arousal	−0.073	0.703	−0.023	0.905	0.360	**0**.**050**
Lubrication/erection	0.120	0.527	−0.027	0.888	0.271	0.148
Ability to reach orgasm	0.190	0.314	0.126	0.507	0.514	**0**.**004**
Satisfaction from orgasm	0.063	0.739	0.168	0.374	0.472	**0**.**008**
Total	0.053	0.782	0.022	0.909	0.450	**0**.**013**

Note. Spearman correlation analysis was used. ASEX: Arizona Sexual Experience Scale. mRs: Modified Rankin Scale, HADS: Hospital Anxiety and Depression Scale (A:Anxiety, D:Depression), SSQOL: Stroke-Specific Quality of Life Scale.

Higher total SSQOL scores were associated with better sexual function across arousal, orgasm, and satisfaction domains (*p* < 0.05). Detailed subscale analysis (Table 5) revealed that Energy and Thinking were the factors most closely associated with sexual health. Energy correlated strongly with arousal (*r* = −0.566, *p* = 0.001) and total ASEX scores (*r* = −0.499, *p* = 0.005). The thinking subscale was significantly linked to almost all sexual domains, including drive, arousal, orgasm, and satisfaction (*p* < 0.05). Furthermore, language impairment was strongly associated with lower arousal (*r* = −0.573, *p* = 0.001). Physical domains, including mobility, self-care, and vision, did not correlate significantly with sexual dysfunction (*p* > 0.05) (Table 5).

**Table 5.** Correlation of ASEX and SSQOL Scale Scores in Stroke Patients.

	ASEX											
	Sex Drive		Arousal		Vaginal Lubrication/Penile Erection		Ability to Reach Orgasm		Satisfaction From Orgasm		Total	
SSQOL	*r*	*p*	*r*	*p*	*r*	*p*	*r*	*p*	*r*	*p*	*r*	*p*
Energy	−0.354	**0**.**055**	−0.566	**0**.**001**	−0.469	**0**.**009**	−0.311	0.095	−0.393	**0**.**032**	−0.499	**0**.**005**
Family roles	−0.006	0.975	−0.157	0.408	−0.103	0.587	−0.212	0.262	−0.195	0.301	−0.132	0.485
Language	−0.275	0.141	−0.573	**0**.**001**	−0.392	**0**.**032**	−0.343	0.064	−0.332	0.073	−0.430	**0**.**018**
Mobility	−0.081	0.670	−0.128	0.502	−0.248	0.186	−0.266	0.155	−0.151	0.427	−0.195	0.302
Mood	−0.206	0.274	−0.222	0.239	−0.233	0.215	−0.462	**0**.**010**	−0.367	**0**.**046**	−0.323	0.081
Personality	−0.162	0.392	−0.242	0.197	−0.175	0.354	−0.525	**0**.**003**	−0.297	0.111	−0.279	0.136
Self care	−0.249	0.184	−0.161	0.397	−0.290	0.120	−0.348	0.060	−0.336	0.070	−0.297	0.111
Social roles	−0.126	0.509	0.097	0.611	−0.192	0.309	−0.231	0.219	−0.072	0.703	−0.131	0.489
Thinking	−0.411	**0**.**024**	−0.456	**0**.**011**	−0.348	0.060	−0.414	**0**.**023**	−0.462	**0**.**010**	−0.481	**0**.**007**
Upper extremity	−0.303	0.104	−0.394	**0**.**031**	−0.219	0.245	−0.345	0.062	−0.247	0.189	−0.316	0.088
Vision	0.060	0.752	0.002	0.990	0.059	0.758	−0.108	0.569	−0.165	0.383	−0.014	0.940
Work/productivity	−0.162	0.391	−0.234	0.212	−0.129	0.498	−0.349	0.059	−0.172	0.365	−0.217	0.250
Total	−0.261	0.164	−0.364	**0**.**048**	−0.333	0.072	−0.491	**0**.**006**	−0.369	**0**.**045**	−0.405	**0**.**026**

Note. Spearman correlation analysis was used. ASEX: Arizona Sexual Experience Scale, SSQOL: Stroke-Specific Quality of Life Scale.

## Discussion

The present study investigated sexual dysfunction and its relationship with functional and psychological state, and quality of life in stroke patients. Our findings indicate higher ASEX scores in patients compared to controls and a substantial decline in subjective sexual satisfaction following the stroke. Notably, the impact of stroke was not limited to overall sexual function; patients demonstrated significantly higher ASEX scores—indicating greater impairment—across all sub-dimensions of sexual function compared to controls. These results align with recent literature reporting sexual issues in 33% to 70% of stroke patients (Geller & Wong, 2025; Latella et al., 2024; Vikan et al., 2021), ranking sexual dysfunction as one of the most common non-motor outcomes following sleep disturbances (Ozkan et al., 2025).

Sexual dysfunction in stroke is multifactorial, stemming from both psychosocial shifts—such as altered self-esteem and role changes—and direct physiological causes. A significant psychological barrier identified in the literature is the “fear of sex,” where patients avoid intimacy due to the misconception that sexual activity might trigger a recurrent stroke (Steiner & IsHak, 2017).

Furthermore, our sample, with a mean age of 49, represents a “young-to-middle-aged” stroke cohort. For individuals in this age group, sexuality is often a central component of identity, social roles, and family planning. Unlike older geriatric populations where sexual decline may be perceived as an age-related expectation, the high prevalence of dysfunction in these younger adults poses a significant threat to their long-term psychological well-being and relational stability (Boller et al., 2015). This underscores the urgency for rehabilitation programs to move beyond simple motor recovery and address the intimate lives of survivors who are still in their productive and socially active years.

Despite its high prevalence, sexual health remains a neglected topic, often shrouded in silence due to the taboos associated with illness and disability. This study's recruitment process itself highlighted this barrier; 20 eligible individuals, primarily women, declined to participate due to discomfort with the topic. In transitional societies, such as Türkiye, where traditional and modern cultural dynamics coexist, discussing sexual health is often considered a taboo, leading patients to feel that such concerns are “inappropriate” or secondary to motor recovery (Abu Aman et al., 2019; Çengelci Özekes et al., 2022). This “silence,” reinforced by both patient hesitancy and a clinician's focus on mobility, leaves the emotional and relational impact of the stroke unaddressed, ultimately diminishing the patient's holistic well-being.

A notable observation in our study was the association between the SSQOL Thinking and Energy domains and self-reported sexual function. While the SSQOL is primarily a health-related quality of life instrument rather than an objective neuropsychological battery, its sub-domains capture important aspects of the patient's everyday experience. Specifically, perceived cognitive challenges, such as self-reported difficulties in processing speed or mental organization, appeared to track closely with lower sexual drive and motivation. Furthermore, the correlation between the Energy sub-domain and arousal suggests that post-stroke fatigue—often experienced as a profound depletion of physical and mental reserves—may act as a significant barrier to intimacy (Hess et al., 2017). Interestingly, within this cohort, sexual dysfunction did not correlate significantly with physical mobility or the severity of disability as measured by the mRS. This highlights the possibility that ‘invisible’ or subjective impairments in energy and cognitive perception may be particularly relevant to sexual health. This pattern aligns with observations by Schneider and Comley-White (2025), who noted that stroke patients with sexual dysfunction report lower quality of life scores across various domains, irrespective of their physical motor recovery.

We observed a relevant discrepancy regarding communication within this cohort: while there was no significant difference in ASEX scores between patients with and without a documented clinical diagnosis of aphasia, a strong correlation existed between the subjective SSQOL Language subdomain and self-reported sexual function. This suggests that a patient's perceived communication difficulty in daily life may track more closely with intimate well-being than the presence of a formal clinical diagnosis alone. Intimacy inherently relies on nuanced communication—such as expressing desires, feelings, and consent. Consequently, subtle fluency challenges or subjective word-finding difficulties, even if not classified as severe clinical aphasia, might contribute to relational frustration and a sense of disconnection (Wolford et al., 2025).

Furthermore, our analysis identified a distinct association between depressive symptoms and sexual dysfunction. The positive correlation between HADS-D scores and total ASEX scores aligns with literature documenting the inhibitory effects of psychological distress on sexual desire. This relationship is often conceptualized as bidirectional; elevated depressive symptoms may track with sexual withdrawal, while the perceived loss of intimacy can potentially exacerbate emotional distress (Greenberg et al., 2017). Interestingly, higher levels of depressive symptoms were linked to lower satisfaction and perceived difficulty reaching orgasm, but did not show a significant correlation with physiological lubrication or erection responses. This observation may suggest that while the physical components of sexual response are mediated by distinct physiological pathways, the subjective experience of pleasure remains closely tied to psychological well-being. However, given the cross-sectional design of our study and the absence of direct neurobiological or vascular tracking, these patterns should be interpreted cautiously as co-occurring clinical associations rather than causal mechanisms.

### Study Limitations

This study has several limitations, including a small, single-center sample and a cross-sectional case-control design that precludes causal inferences regarding the relationships between stroke and sexual function. The COVID-19 pandemic also hampered data collection by limiting access to a sufficient number of patients, and research data was collected between October 2020 and April 2023. We did not assess pre-stroke baseline sexual function, introducing potential recall bias through retrospective measurements. Furthermore, while we utilized an age- and gender-matched control group, the control group tended toward a younger mean age (*p* = 0.057). Although this difference did not reach statistical significance, the potential influence of age on sexual function should be considered when interpreting the comparative results. Additionally, our standard screening tools carry inherent conceptual boundaries: the ASEX lacks coverage of intimacy and partner engagement; the SSQOL captures subjective perceptions of quality of life domains rather than objective neurobiological performance; and the HADS evaluates sub-clinical symptom severity rather than formal psychiatric diagnoses. Lastly, our strict exclusion of individuals with significant clinical anxiety or depression preserves internal validity but restricts the cohort's psychological variance, while the low physical disability profile limits generalizability to broader stroke populations. Nevertheless, these preliminary patterns offer a transparent, realistic foundation for future multi-center longitudinal research.

## Conclusion

In conclusion, this study suggests that sexual dysfunction represents a multifaceted challenge for young-to-middle-aged stroke patients. Our findings indicate that self-reported sexual health is closely tied to specific quality of life domains—namely subjective energy, communication, and emotional well-being—showing a more notable association within this cohort than objective physical mobility. The observed frequency of dysfunction, even in patients with low physical disability underscores the possibility that post-stroke sexual health may be affected more significantly than is typically recognized in routine clinical screening. Given that these preliminary patterns stem from a productively and socially active cohort, considering sexual health within a holistic rehabilitation framework appears warranted. Proactively exploring these quality of life dynamics during neurorehabilitation could potentially support long-term well-being. Ultimately, given our design's inherent boundaries, future multi-center longitudinal research is necessary to further examine the complex relationships between longitudinal quality of life recovery and long-term sexual health outcomes.
